# A phase 1/2a, dose-escalation, safety, pharmacokinetic, and preliminary efficacy study of intraperitoneal administration of BC-819 (H19-DTA) in subjects with recurrent ovarian/peritoneal cancer

**DOI:** 10.1007/s00404-017-4293-0

**Published:** 2017-02-03

**Authors:** Ofer Lavie, David Edelman, Tally Levy, Ami Fishman, Ayala Hubert, Yakir Segev, Eli Raveh, Michal Gilon, Avraham Hochberg

**Affiliations:** 1 Department of Obstetrics and Gynecology Carmel Medical Center, The Rappaport Faculty of Medicine, Technion, Haifa, Israel; 2 Sharett Institute of Oncology, Hadassah-Hebrew University Medical Center, Jerusalem, Israel; 3 Department of Obstetrics and Gynecology, The Edith Wolfson Medical Center-Holon, Sackler School of Medicine, Tel-Aviv University, Tel-Aviv, Israel; 4 Department of Gynecology and Obstetrics, Meir Hospital Kfar-Saba, Sackler School of Medicine, Tel-Aviv University, Tel-Aviv, Israel; 5 BioCancell Therapeutics Ltd, Jerusalem, Israel; 6 The Department of Biological Chemistry, Institute of Life Sciences, The Hebrew University of Jerusalem, Jerusalem, Israel

**Keywords:** Intraperitoneal, BC-819 (H19-DTA), Recurrent ovarian/peritoneal cancer

## Abstract

**Background:**

H19 is a paternally imprinted, oncofetal gene expressed in various embryonic tissues and in 85% of the ovarian tumors. H19-DTA (BC-819) is a DNA plasmid that drives the expression of the diphtheria toxin gene under the regulation of the H19 promoter sequence and therefore is a potential treatment for various tumors that overexpress the H19 gene, among them—ovarian cancer.

**Objective:**

To assess the safety and efficacy of intra-peritoneal (IP) instillations of H19-DTA (BC-819) plasmid in treating ovarian/peritoneal cancer patients with advanced recurrent disease.

**Methods:**

A phase 1–2A multi-centric trial included 14 eligible patients who were either platinum-refractory or platinum-resistant with positive H19 expression. Patients were treated IP with escalating weekly doses of BC-819 for a maximum of 6–9 weeks. Dose-limiting toxicities (DLT) were assessed after the first course of treatment for each patient and each subsequent cohort was enrolled once each subject had completed the first course of treatment and its 4-week follow-up period. The occurrence of adverse events (AEs) and response to treatment were assessed after the induction course and then periodically.

**Results:**

During the study, no DLTs were observed. Only 5 grade 1 and 2 AEs, which occurred in 4 patients were considered as possibly related to BC-819. The best tumor response seen was stable disease. Median survivals of 3.2, 5.3 and 6.5 months were observed for the 60, 120 and 240 mg cohorts, respectively.

**Conclusions:**

BC-819 can be considered safe and well tolerated in intraperitoneal doses up to 240 mg. Hybridization of intraperitoneal chemotherapy with the biological treatment of BC-819 should be further evaluated in phase 2 and 3 studies.

## Introduction

Ovarian cancer is a biologically aggressive cancer with exceptionally high mortality rate, making it the fifth most common causes of death from malignancy in women [1]. In the United States, ovarian cancer is the seventh most common cancer in women accounting for almost one-third of invasive malignancies of the female genital organs and has remained the leading cause of death from gynecological cancers with nearly 22,000 new cases and 15,460 deaths in 2011 [1].

The majority of patients with ovarian cancer will have advanced-stage disease at initial diagnosis and this is intimately linked with the poor prognosis of the disease [2, 3].

Most patients with advanced stage disease will experience relapse, and only 20–25% of patients can be expected to be long-term survivors, despite a good response to primary treatment [4].

The primary intervention for advanced stage ovarian cancer is debulking surgery followed by chemotherapy with platinum-based analogues and paclitaxel and/or neoadjuvant chemotherapy followed by debulking surgery and adjuvant chemotherapy [3, 5–7].

Despite the improved median overall survival in patients with such chemotherapy regimens, relapse still occurs in the majority of those with advanced disease, and only 10–30 % of such patients have long-term survival [4–6, 8, 9].

Ovarian cancer is a disease that initially spreads throughout the abdominal cavity, although in some cases a pleural effusion or extraperitoneal spread can be detected [10]. The mortality associated with ovarian cancer is primarily due to dissemination of the disease within the peritoneal cavity due to the absence of early diagnostic symptoms. When the peritoneal cavity is involved, conventional therapies such as surgery and chemotherapy in most of the cases fail to provide long-term cure [11].

Three randomized prospective studies of IP chemotherapy [12–14] documented an advantage in overall survival for patients receiving chemotherapy in the IP arm, and despite a significant short-term quality of life deterioration in the IP, these prospective studies suggested a possible advantage for tumor lysis through this drug delivery route.

H19 is a paternally imprinted, oncofetal gene that encodes a ribonucleic acid (RNA), with no protein product, which acts as a “riboregulator”. It is upregulated in tumor cells and promotes cancer progression, angiogenesis, and metastasis [15–17]. BC-819 (formerly, DTA-H19) is a double-stranded deoxyribonucleic acid (DNA) plasmid, 4,560 base pairs (bp) in length, carrying the gene for the diphtheria toxin A (DT-A) chain under the regulation of the 814 bp 5′ flanking region of the H19 promoter sequence. DT-A chain expression is triggered by the presence of transcription factors that are upregulated in tumor cells. The selective initiation of toxin expression results in selective tumor cell destruction via inhibition of protein synthesis in the tumor cell, enabling highly targeted cancer treatment. This therapy is determined by tumor tissue/cell screening for H19 RNA expression in order to select the appropriate patient population and to ensure success of treatment.

Previous non-clinical studies in animals showed that BC-819 inhibited tumor cell growth in a heterotopic nude mouse ovarian cancer model [18] and slowed tumor cell growth in a nude mouse ovarian cancer ascites model (unpublished data). In humans, BC-819 was administered in bladder [19] and pancreatic [20] carcinoma and to an ovarian cancer patient under a compassionate protocol [21].

The intraperitoneal [IP] administration route allows reduced systemic exposure and its possible toxic effects [22] together with higher availability of the drug over time. The IP administration of BC-819 has the potential to reach ascites tumor cells, deliver its intracellular toxin and selectively destroy tumor cells without targeting normal tissues, and thus helping to control this aspect of ovarian cancer.

The aim of the current study was to determine the safety, tolerability, PK, preliminary efficacy and quality of life (QoL) of BC-819 administered IP in subjects with advanced stage ovarian cancer. The study’s primary endpoint was to assess the DLTs of BC-819 and its maximum tolerated dose (MTD) in this patient population.

### Study design

This was a Phase 1/2a, open label, dose-escalation, repeat dose study in 14 subjects with recurrent, platinum-resistant advanced stage ovarian cancer or primary peritoneal carcinoma. Following a screening period of up to 6 weeks, subjects were enrolled in 3 cohorts. Subjects in the first cohort received, IP, an absolute dose of 60 mg of BC-819 and sequential cohorts received escalating doses of BC-819 (120 mg in the second cohort, and 240 mg in the third cohort, absolute doses).

The dose and schedule of BC-819 administration was based in part on preclinical animal-efficacy studies, in which it was shown that intraperitoneal injection of the plasmid significantly reduced the growth rate of ovarian carcinoma and reduces the amount if ascites accumulation as compared with the control group in an orthotopic animal model for ovarian cancer.

BC-819 was supplied as a frozen liquid formulated to contain 4 mg/mL of plasmid DNA. Vials were thawed and diluted to a total volume of 500 mL with sterile 0.9% preservative-free saline. BC-819 was administered intraperitoneally via a fully implantable port attached to a single-lumen catheter which was placed SC on the left inferior thorax at the mid-clavicular line above ribs number 9–10. Subjects were assessed by CT or positron emission tomography/CT at screening and in the final week of every treatment course follow-up. No additional surgeries (secondary debulking nor palliative surgery) were performed in any of the patients along the study period.

## Patients and methods

### Patient selection

Patients were recruited from 4 gynecological oncology centers in Israel after the protocol was reviewed and approved by the research ethics committee of each participating site. Written informed consent was obtained from each patient at the time of enrollment. After obtaining informed consent, patients were screened over a week period for medical history, prior cancer treatments and medication use, physical examination, electrocardiogram, hematology, blood chemistries, coagulation markers, tumor markers, urinalysis, Karnofsky performance status, vital signs, height and weight, tumor biopsy(/ies) for histopathology and ISH (in situ hybridization) for H19 gene expression, radiological assessments including chest X-ray, chest, abdominal and pelvic computerized tomography (CT), and/or abdominal CT/positron emission tomography, and other scans as clinically indicated (magnetic resonance imaging, brain CT, bone scan). Subjects were assigned to cohorts sequentially when determined to be eligible for the study. The first eight eligible subjects were assigned to cohort no. 1; 60 mg IP BC-819 weekly for 3 weeks, one week rest, then repeat for 2 more courses, except for two patients that were treated according to a revised protocol, due to FDA recommendation; those two patients were treated weekly for 3 weeks, followed by 4 weeks rest and safety follow-up for DLTs (instead of 1 week safety follow-up), and then repeating one additional course. The FDA recommendations arose in a discussion following what was analyzed as drug-unrelated deaths that occurred during the study, and intended to change the study design and, respectively, revise the protocol so as to allow completion of treatment courses and make it more consistent with a Phase 1 safety study design. Once a subject had completed one course of treatment with 4 weeks of follow-up with no progressive disease or toxicity warranting discontinuation, he was considered evaluable for the assessment of DLTs.

The second three eligible subjects were assigned to cohort no. 2; 120 mg IP BC-819 weekly for 3 weeks, 4 weeks rest, then if possible repeat for one additional course, and the last three eligible subjects were assigned to cohort no. 3 (240 mg IP BC-819 weekly for 3 weeks, 4 weeks rest, then if possible repeat for one additional course). The 120 and 240 mg cohorts adhered to the revised protocol.

As this was a multi-site study, dose escalation and enrollment were carefully coordinated between the study sites.

Potential study subjects included those with a histological advanced stage recurrent ovarian cancer or primary peritoneal carcinoma who had either platinum-refractory disease or platinum-resistant recurrent disease.

Patients also had a Karnofsky performance status of ≥70% with acceptable hematopoietic parameters, liver and renal function tests. A minimum of 30 days from the last active treatment was required before screening. Patients agreed to refrain from any concurrent chemotherapy, hormonal therapy, radiotherapy, immunotherapy or any other type of therapy for the treatment of cancer while on this protocol.

### AEs evaluation

AEs included events reported by the subject, as well as clinically significant abnormal findings on clinical examination or laboratory evaluation. A new illness, symptom, sign or clinically significant clinical laboratory abnormality or worsening of a pre-existing condition or abnormality was considered an AE. In addition, abnormal laboratory values that met the criteria for an AE in accordance with the Common Terminology Criteria for Adverse Events (CTCAE), even if not considered clinically significant were reported as an AE. The CTCAE dictated also the severity grading of each AE. Stable chronic conditions, such as arthritis, which were present prior to enrollment and did not worsen, were not considered AEs.

Events were regarded generally “related” to BC-819 in case their time relationship to BC-819 treatment was not incompatible or making a casual connection improbable.

### Statistical analysis

An analysis of the study data was performed when all of the subjects completed the study through at least week 4. The intention-to-treat and safety population were defined as all subjects who received the first intraperitoneal administration of the investigational product. The per protocol population included subjects who met the study inclusion and exclusion criteria, received all three study treatments with the investigational product (i.e., a complete treatment course) and had a follow-up disease assessment to examine the tumor response. Descriptive statistics for continuous variables, including the median and range, and for categorical variables, including the count and percent, were used to describe the study data.

## Results

A total of 14 Caucasian female subjects fulfilled the inclusion criteria and enrolled into the study. Baseline characteristics of the patients participating in the study are shown in Table 1: the mean age of the study population was 59.6 ± 9.8 years (range 38.0–75.0 years). All 14 subjects had stage 3-C ovarian cancer when first diagnosed. The mean and median times from disease diagnosis were 3.2 ± 2.1 years and 2.5 years (range 0.8–7.1 years), respectively. All subjects were heavily pretreated with chemotherapy prior to enrollment (mean of 4 courses, range 1–10).

**Table 1:** Baseline characteristics of the study population

Parameter	BC-819 60 mg (*N* = 8)	BC-819 120 mg (*N* = 3)	BC-819 240 mg (*N* = 3)	All (*N* = 14)
Mean age (years) ± SD	60.9 ± 12.0	61.3 ± 3.1 (3)	54.3 ± 7.6	59.6 ± 9.8
Mean height (cm) ± SD	158.6 ± 7.4	166.7 ± 1.5	157.3 ± 6.1	160.1 ± 7.0
Mean weight (kg) ± SD	71.4 ± 11.4	69.3 ± 5.8	62.9 ± 22.0	69.1 ± 12.7
Karnofsky performance status score ± SD	83.8 ± 7.4	80.0 ± 0.0	93.3 ± 5.8	85.0 ± 7.6
Ascites evaluation				
Grade 1 ascites, *n* (%)	0 (0)	0 (0)	0 (0)	0
Grade 2 ascites, *n* (%)	1 (12.5)	2 (66.7)	2 (66.7)	5 (35.7)
Grade 3 ascites, *n* (%)	5 (62.5)	1 (33.3)	0	6 (42.9)
Without ascites	2 (25)	0 (0)	1 (33.3)	3 (21.4)

Of the 14 subjects who entered this study 8 subjects were enrolled into the 60 mg cohort, 3 subjects to the 120 mg cohort and 3 subjects to the 240 mg cohort (Table 2).

**Table 2:** Doses of BC-819 in each study cohort (PP population)

Cohort number	Number of patients	Dose	
1	6	Initial protocol	60 mg IP weekly for 3 weeks, 1 week rest, then repeat for 2 more courses (if possible)
	2	Revised protocol	60 mg IP weekly for 3 weeks, 4 week rest, then repeat for 1 more course (if possible)
2	3		120 mg IP weekly for 3 weeks, 4 week rest, then repeat for 1 more course (if possible)
3	3		240 mg IP weekly for 3 weeks, 4 week rest, then repeat for 1 more course (if possible)

Of the 8 subjects enrolled into the 60 mg cohort, 2 subjects completed the study, while 5 subjects withdrew prematurely due to overall clinical deterioration (2 patients) or requested to withdraw prematurely (3 patients, of them one had also a serious infection and overall clinical deterioration) and one subject discontinued due to tumor progression per CT, clinical assessment and CA-125 elevation. Of the 3 subjects who were enrolled into the 120 mg cohort, 1 subject completed the study and 2 withdrew prematurely due to overall clinical deterioration, concurrent illness, and disease progression. All three subjects enrolled into the 240 mg cohort withdrew prematurely from the study due to overall clinical deterioration and disease progression.

### Exposure to treatment

The maximal exposure possible for each subject was 6–9 absolute doses of BC-819 60 mg (2–3 courses of 1 infusion/week for 3 weeks). On average, the patients in the 60 mg cohort were exposed to 270 ± 126 mg of BC-819, the patients in the 120 mg cohort were exposed to 480 ± 204 mg of BC-819, and the patients in the 240 mg cohort were exposed to 720 ± 0.0 mg of BC-819.

### Pharmacokinetics evaluation during treatment

There was a high variability in systemic (venous) PK parameters among patients, and plasma exposure increases measured by C_max_ (maximal concentration) and AUC_inf_ the calculated integral of the concentration–time curve, extrapolated to infinity were not proportional with dose.

**Fig. 1:**
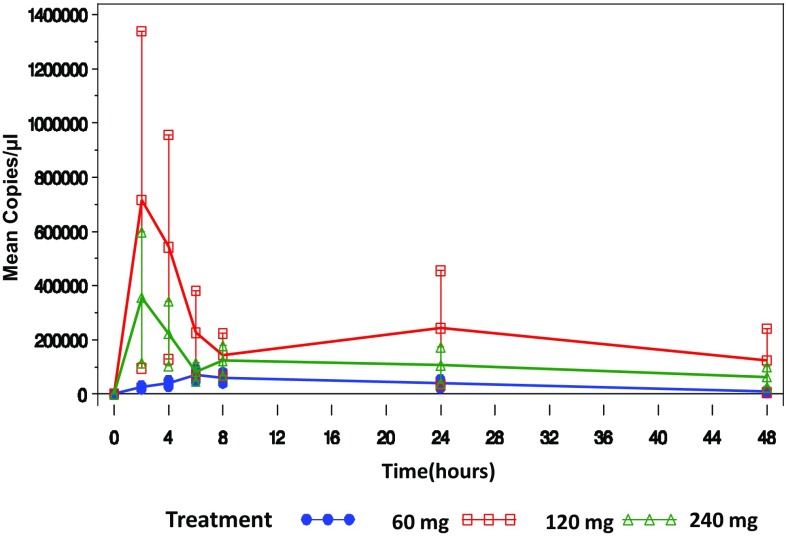
Mean copies/µl blood ± SE by treatment and time point

C_max_ and AUC_inf_ were higher in the 120 mg cohort than those observed in the 60 mg but also higher than those observed in the 240 mg cohort (Fig. 1). This may indicate altered absorption from the peritoneum due to the disease. Two peaks of plasmid were observed in the blood in 8/11 of the patients. The first peak was observed 2–8 h after BC-819 administration, and the second peak was observed 6–48 h after plasmid administration. The peaks can be explained by the fact that some of the plasmid may be transferred to the bloodstream from the peritoneum, which might be indicated by the first peak, and then transferred through the lymphatic system into the venous bloodstream, which might be represented by the later second peak in the graphs [23]. Plasmid plasma levels remained quantifiable for up to 48 h in all cohorts (last PK measurement). Terminal elimination half-life was proportional with dose.

**Fig. 2:**
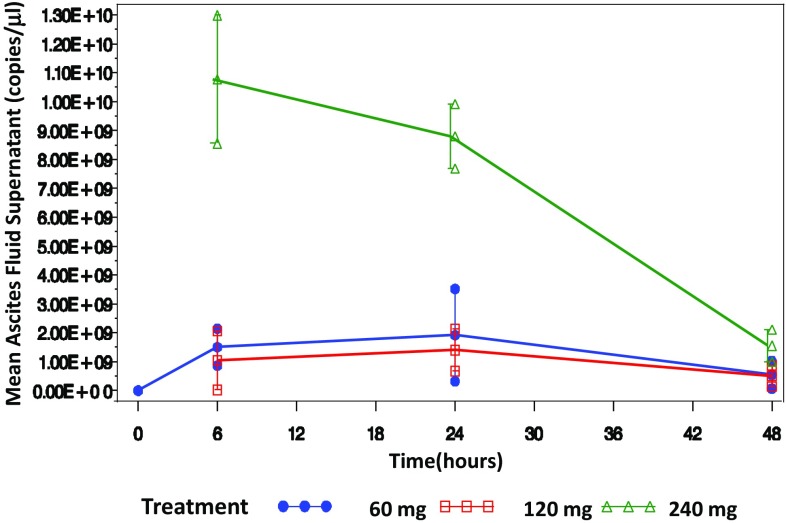
Mean copies/[µl of fluid] ±SE by treatment and time point in ascites fluid supernatant

The PK parameters for ascites fluid cells and for ascites fluid supernatant are summarized in Tables 3 and 4, respectively, and the average ascites supernatant PK profiles for each dose are presented in Fig. 2.

**Table 3:** Summary of main PK parameters by treatment in the cellular fraction of the ascites fluid

Parameter	BC-819 60 mg Mean ± SD *N* = 8	BC-819 120 mg Mean ± SD *N* = 3	BC-819 240 mg Mean ± SD *N* = 2
C_max_ (copies/μL)	6.32E8 ± 7.49E8	428E8 ± 738E8	9.37E9 ± 116E8
T_max_ (h)	12.8 ± 9.3	20.0 ± 24.2	6.0 ± 0.0
T 1/2 (h)	10.0 ± 6.9	13.2 ± 5.3 (*n* = 2)	6.4 ± 0.7
AUC last (copies/µl × h)	129E8 ± 179E8	517E9 ± 887E9	122E9 ± 154E9
AUC inf (copies/µl × h)	137E8 ± 18E9	5.05E9* ± 5.78E9 (*n* = 2)	123E9 ± 155E9

*SD* standard deviation

*N = 2

**Table 4:** Summary of main PK parameters by treatment in the ascites fluid supernatant

Parameter	BC-819 60 mg Mean ± SD *N* = 8	BC-819 120 mg Mean ± SD *N* = 3	BC-819 240 mg Mean ± SD *N* = 2
C_max_ (copies/µL)	2.65E9 ± 3.94E9	1.6E9 ± 1.52E9	108E8 ± 3.14E9
T_max_ (h)	33.0 ± 55.3	12.0 ± 10.4	6.0 ± 0.0
T 1/2 (h)	11.5 ± 6.7 (*N* = 4)	60.7 ± 71.3	15.6 ± 6.5
AUC last (copies/µl × h)	568E8 ± 114E9 (*N* = 7)	45E9 ± 47E9	301E9 ± 518E8
AUC inf (copies/µl × hours)	105E9 ± 174E9(*N* = 4)	66.3E9 ± 73.8E9	339E9 ± 193E8

After a single IP dose of BC-819, plasmid levels were still detectable in all cohorts after 48 h (in one subject in the 60 mg cohort, plasmid levels were still detectable after 1 week). No dose proportionality in PK parameters could be detected due to high variability in PK parameters among patients.

In ascites fluid supernatant, the mean PK parameters of the 240 mg cohort were higher than those of the other cohorts, while in the blood these parameters were lower than those of the 120 mg cohort, indicating a different behavior in both compartments.

For mean number of copies of plasmid in ascites fluid cells, mean AUClast (the calculated integral of the concentration–time curve, from time 0 to last measurable concentration) is based on three patients and mean AUCinf is based on two patients only, as one patient had no AUCinf estimation. This patient had an extremely high plasmid count per 0.1 µg cellular DNA, and the level went up at 48 h, making it impossible to calculate a T1/2 for that patient and skewing the AUC for that cohort.

### Adverse events (AE)

All 14 treated subjects reported a total of 148 AEs during the study. Of these, 99 AEs occurred in the 60 mg cohort, 28 AEs in the 120 mg cohort, and 21 in the 240 mg cohort. Five possibly drug-related AEs were reported: 4 in the 60 mg cohort and 1 in the 120 mg cohort (Table 5). No drug-related AEs were reported in the 240 mg cohort (all possibly drug-related AEs were grade 1 or 2). There was not a dominant AE which was related to one of the essential body systems.

**Table 5:** Possibly drug-related AEs

	BC-819 60 mg		BC-819 120 mg		All	
	Patients, *N* (%)	Events, *N*	Patients, *N* (%)	Events, *N*	Patients, *N* (%)	Events, *N*
Cardiac disorders						
Dizziness	1 (12.5)	1			1 (7.1)	1
Gastrointestinal disorders						
Nausea	1 (12.5)	1			1 (7.1)	1
General disorders and administration site conditions						
Asthenia			1 (33.3)	1	1 (7.1)	1
Pyrexia	1 (12.5)	1			1 (7.1)	1
Skin and subcutaneous tissue disorders						
Erythema	1 (12.5)	1			1 (7.1)	1

Fifteen (15) treatment-emergent SAEs were reported in 7 treated patients. Of these, 12 SAEs were reported by 6 patients in the 60 mg cohort, and 3 were reported by 1 patient in the 120 mg cohort. None of the SAEs were related to the study drug.

The incidence of AEs occurring in two or more patients is shown in Table 6. The most common adverse events reported were vomiting and a decrease in calcium blood levels (in 50% of all patients), asthenia and activated partial thromboplastin time prolonged (42.9% each), and blood albumin decreased (35.7%). All 14 (100%) patients reported laboratory AEs. General disorders and administration site conditions were reported by 10 patients (71.4%) and gastrointestinal disorders were reported by 9 patients (64.3%). In general, there was no evidence of a dose–response effect for frequency of individual adverse events. The only adverse events occurring more frequently in the 240 mg group than in the other two treatment groups were [increased prothrombin time]/[international normalized ratio] and decreased white blood cell count.

**Table 6:** Summary of adverse events occurring in two or more patients in any cohort

System organ class	No. patients reporting AEs (%)			
	BC-819 60 mg	BC-819 120 mg	BC-819 240 mg	All
Cardiac disorders	1 (12.5)	2 (66.7)		3 (21.4)
Gastrointestinal disorders	6 (75.0)	2 (66.7)	1 (33.3)	9 (64.3)
Constipation	2 (25.0)	–	1 (33.3)	3 (21.4)
Diarrhea	3 (37.5)	1 (33.3)	–	4 (28.6)
Dyspepsia	1 (12.5)	–	1 (33.3)	2 (14.3)
Vomiting	6 (75.0)	1 (33.3)	–	7 (50.0)
General disorders and administration site conditions	5 (62.5)	3 (100.0)	2 (66.7)	10 (71.4)
Asthenia	4 (50.0)	2 (66.7)	–	6 (42.9)
General physical health deterioration	2 (25.0)	–	–	2 (14.3)
Infections and infestations	1 (12.5)	1 (33.3)		2 (14.3)
Injury, poisoning and procedural complications	3 (37.5)			3 (21.4)
Fall	2 (25.0)	–	–	2 (14.3)
Investigations	8 (100)	3 (100)	3 (100)	14 (100)
Activated partial thromboplastin time prolonged	4 (50.0)	1 (33.3)	1 (33.3)	6 (42.9)
Alanine aminotransferase increased	3 (37.5)	–	–	3 (21.4)
Aspartate aminotransferase increased	4 (50.0)	–	–	4 (28.6)
Blood albumin decreased	2 (25.0)	2 (66.7)	1 (33.3)	5 (35.7)
Blood calcium decreased	3 (37.5)	3 (100.0)	1 (33.3)	7 (50.0)
Blood potassium increased	3 (37.5)	1 (33.3)	–	4 (28.6)
Hemoglobin decreased	3 (37.5)	–	1 (33.3)	4 (28.6)
International normalized ratio increased	2 (25.0)	–	2 (66.7)	4 (28.6)
White blood cell count decreased	–	–	2 (66.7)	2 (14.3)
Metabolism and nutrition disorders	2 (25.0)	1 (33.3)		3 (21.4)
Hypocalcemia	2 (25.0)	1 (33.3)	–	3 (21.4)
Nervous system disorders	2 (25.0)		1 (33.3)	3 (21.4)
Headache	2 (25.0)	–	1 (33.3)	3 (21.4)
Renal and urinary disorders	4 (50.0)		1 (33.3)	5 (35.7)
Urinary tract infection	3 (37.5)	–	–	3 (21.4)
Respiratory, thoracic and mediastinal disorders	1 (12.5)	2 (66.7)		3 (21.4)
Dyspnoea	1 (12.5)	2 (66.7)	–	3 (21.4)
Skin and subcutaneous tissue disorders	2 (25.0)	1 (33.3)		3 (21.4)
Erythema	1 (12.5)	1 (33.3)	–	2 (14.3)
Rash	1 (12.5)	1 (33.3)	–	2 (14.3)
Vascular disorders	1 (12.5)	1 (33.3)		2 (14.3)

Thirteen (13) grade 3 AEs were reported during the study; 9 in the 60 mg cohort and 4 in the 120 mg cohort. One grade 4 AE (pulmonary embolism) was reported in the 120 mg cohort. No grade 3 or 4 AEs were reported by more than one patient, and none were reported in the 240 mg cohort. None of the grade 3 or 4 AEs were considered related to the study drug (Table 7).

**Table 7:** Display of grade 3 and 4 AEs by MedDRA® Categories

Preferred term	Grade	BC-819 60 mg		BC-819 120 mg		All	
		Patients, *N* (%)	Events, *N*	Patients, *N* (%)	Events, *N*	Patients, *N* (%)	Events, *N*
Blood and lymphatic system disorder							
Anemia	3	1 (12.5)	1			1 (7.1)	1
Gastrointestinal disorders							
Abdominal pain	3	1 (12.5)	2			1 (7.1)	2
Vomiting	3	1 (12.5)	1			1 (7.1)	1
General disorders and administration site conditions							
Asthenia	3	1 (12.5)	1			1 (7.1)	1
Device occlusion	3			1 (33.3)	1	1 (7.1)	1
General physical health deterioration	3	1 (12.5)	1			1 (7.1)	1
Injury, poisoning and procedural complications							
Procedural pain	3	1 (12.5)	1			1 (7.1)	1
Investigations							
Blood calcium decreased	3			1 (33.3)	1	1 (7.1)	1
Hemoglobin decreased	3	1 (12.5)	1			1 (7.1)	1
Hepatic enzyme increased	3	1 (12.5)	1			1 (7.1)	1
Neoplasms benign, malignant and unspecified (incl cysts and polyps)							
Neoplasm malignant	3	1 (12.5)	1			1 (7.1)	1
Respiratory, thoracic and mediastinal disorders							
Dyspnoea	3			1 (33.3)	1	1 (7.1)	1
Vascular disorders							
Pulmonary embolism	4			1 (33.3)	1	1 (7.1)	1

### Response to treatment

The efficacy endpoints of the study were ascites response, solid measurable disease, survival and quality of life. Although serum was collected for quantitative measurement of CA-125 (at screening and in the final week of every treatment course follow-up), the fact that this marker’s levels may rise when injecting into the peritoneum [24], disqualified this measure as part of the tumor response measurements.

Ascites response was assessed by ultrasound and by numbers and volumes of paracenteses at various times during treatment. The best ascites-related response that was observed during the study was stable disease with persistence of ascites (i.e., incomplete response/stable disease).

Response outcomes were applied according to RECIST criteria for solid tumors [13]. The best overall response for solid tumor masses was stable disease i.e., insufficient shrinkage to qualify for partial response (at least 30% decreased in the longest diameter) and insufficient increase to qualify for progressive disease (at least a 20% increase in the longest diameter of the target lesion).

Table 8 shows the best response (measured at least 6 weeks after the start of treatment) by treatment group, for solid tumor masses. Overall, 4 patients (31%) had stable disease at the first assessment. There were no complete or partial responses.

**Table 8:** Tumor response in the ITT **(intent to treat)** population

	BC-819 dose, mg			All patients
	60	120	240	
*N*	7 (87.5%)*	3 (100)	3 (100)	13 (92.9)*
Stable disease	2 (29)	2 (67)		4 (31)
Progressive disease	2 (29)	1 (33)	3 (100)	6 (46)
Not evaluable	3 (43)			3 (23)

*One patient was not assessed for tumor response

**Fig. 3:**
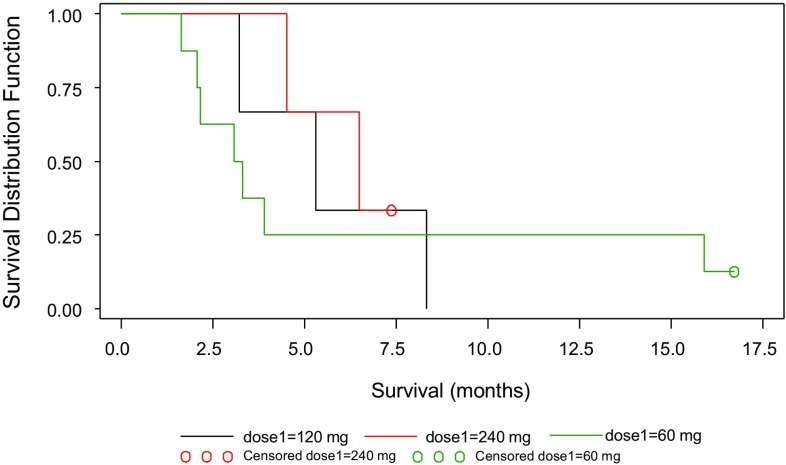
Kaplan–Meier curve for survival—ITT population

Overall survival, defined as the time from the start of treatment until the subject died, and estimated by Kaplan Meier curves for the **intent to treat (**ITT) (Fig. 3) and for the **per protocol** (PP) population, is presented in Table 9.

**Table 9:** Overall survival in the ITT and the PP populations

	Population	Survival (months)	
		ITT	PP
60 mg	N	8	5
	Median (range)	3.2+ (1.6–16.7+)	3.9+ (3.1–16.7+)
120 mg	N	3	3
	Median (range)	5.3 (3.2–8.3)	5.3 (3.2–8.3)
240	N	3	3
	Median (range)	6.5+ (4.5–7.4+)	6.5+ (4.5–7.4+)
All	N	14	11
	Median (range)	4.2+ (1.6–16.7+)	5.3+ (3.1–16.7+)

*SD* standard deviation, *ITT* intention to treat, *PP* per protocol; +censored data

Survival data for this cohort was denoted by median overall survival in the ITT population which was estimated at 4.2+ months (range 1.6–16.7 months), and 5.3+ months in the PP population (range 3.1–16.7 months). There appears to be a dose–response relationship for survival (Fig. 3) with median survival 3.2, 5.3 and 6.5 months for the 60, 120 and 240 mg ITT cohorts, respectively. These observations should be evaluated with caution due to the small cohort sizes, differences with regard to overall performance status and the possibility of selection bias towards healthier patients as the trial progressed.

### Quality of life

Composite scores and subscale scores from the FACT-O were used to assess QoL outcomes. Although assessment of QoL showed an overall worsening for the whole patient population treated (i.e., decrease in total score), assessment of FACT-O composite scores by visit showed an improvement in QoL of the 60 mg cohort on visits 6, 7 and 11 (ITT population) and on visits 5, 6, 7 and 11 (PP population); an improvement in QoL on visits 4, 8, 11, 12 and 13 in the 120 mg cohort (PP and ITT populations); and an increase in total score on visit 4 in the 240 mg cohort (PP and ITT populations).

Due to the small number of subjects in each cohort, the change in QoL scores from baseline could not be assessed statistically.

### Pain scale scores

An increase in pain scale scores was observed in the 60 mg cohort on visits 4, 10, 11, 14 and 15. A decrease in pain scale scores was observed in the 60 mg cohort on visits 1 and 5, and in both the 120 and the 240 mg cohorts on visit 5.

## Discussion

The aim of this study was to assess the pharmacokinetics, and preliminary efficacy of BC-819 administered IP in subjects with recurrent, platinum-resistant advanced stage heavily pretreated ovarian cancer or primary peritoneal carcinoma.

Fourteen patients, who were heavily pretreated with chemotherapy prior to enrollment, participated in the study.

Three dose levels of BC-819 were assessed: 60, 120, and 240 mg. In all dosages, plasmids were detected in blood as well as in the cellular and fluid fractions of the ascites 2 days post-administration and in some cases even more. We have already shown elsewhere that BC-819 administration IP to ovarian cancer patient resulted in RNA presence of DTA in ascites cells [21]. The primary objectives of this study were to identify any dose-limiting toxicity and to determine the maximal tolerated dose of BC-819. During the study no dose-limiting toxicity was observed and therefore no maximal tolerated dose could be established. The highest dose administered was 240 mg of BC-819 which can therefore be considered as safe and well tolerated.

The majority of AEs reported were considered to be related to the patients’ underlying advanced ovarian cancer, and/or general clinical deterioration due to disease progression. Only five grade 1 and 2 AEs were considered to be possibly related to BC-819 and no serious AEs were considered to be related to the investigational drug. One serious AE was considered related to the study procedure. These findings suggest that BC-819 is a safe and well-tolerated novel technology for patients with platinum-resistant advanced stage heavily pretreated ovarian cancer or primary peritoneal carcinoma.

The secondary objectives of the study included the determination of the overall survival distribution. Overall survival seen in this study is in accordance with other studies which reported a range of 6.3–15 months for median overall survival in heavily pretreated patients with recurrent platinum-resistant ovarian cancer [25–30].

The best ascites-related response that was observed during the study was stable disease with persistence of ascites. The best response for solid tumors was stable disease (i.e., insufficient shrinkage to qualify for partial response and insufficient increase to qualify for progressive disease). These findings may suggest a potential response of less advanced ovarian/peritoneal tumors to BC-819.

PK analysis indicated that BC-819 remains in ascites fluid cells and supernatant for at least 48 h. It is absorbed into the blood and remains quantifiable for at least 48 h (the last PK measurement). Two peaks of plasmid in the blood were observed in 8 of the patients; this may indicate that some of the plasmid is transferred to the bloodstream from the peritoneum (first peak), and then it is transferred through the lymphatic system into the venous bloodstream (second peak). A similar pattern was observed in the compassionate use ovarian cancer patient treated with BC-819. As there was high variability among patients in the PK parameters measured, no conclusions can be made regarding the relationship between drug dose and PK of BC-819.

### Determination of the QoL of the subjects receiving BC-819

QoL, as assessed by the FACT-O scale, showed overall worsening of the QoL score; however, improvement in QoL was observed on some study visits. The change in QoL could not be evaluated statistically due to the small number of subjects in each cohort. Since all participating patients in the trial had ovarian or peritoneal tumors expressing H19, the question arises: why some tumors, expressing H19, did not respond?

The preliminary results of this phase1/2 study can be explained by several mechanisms:

1. Patients with more advanced or aggressive disease have a tendency for being “Non-responding” patients suggesting that in early disease the BC-819 will produce a better tumor response rate.
2. There could be kinetic obstacle that prohibits long enough exposure of malignant and premalignant cells to the plasmid.
3. In pre-heavily treated tumor cells the H19 gene is not active, thus no toxin is released in the tumor cells, suggesting the failure of BC-819 in ablating the tumor marker.

In the era where intraperitoneal chemotherapy and biological treatments for advanced stage ovarian and peritoneal carcinoma are suggesting a significant advantage in the disease-free interval and in overall survival, the development of a biological anti-tumor DNA-based therapy and the use of a combination of biological agents with conventional chemotherapy with an IV and IP approach may offer a promising advantage in the response rate or in the overall survival of these patients.

In this study a tumor-selective promoter was used in conjunction with a cytotoxic gene to achieve targeted tumor cell destruction. The plasmid BC-819 has advantages over viral vectors including lack of immunogenicity and cytotoxicity allowing repeated treatments.

Taken together, the data of this study suggest that:

1. BC-819 can be considered safe and well tolerated in intraperitoneal doses up to 240 mg.
2. BC-819 given locally in combination with systemic chemotherapy may provide additional therapeutic benefit for the treatment of ovarian or peritoneal cancer.
3. The hybridization of intraperitoneal chemotherapy with the biological treatment of BC-819 should be further evaluated in phase 2 and 3 studies.

This study confirmed the excellent safety profile of BC-819 with a low rate of adverse effects and no grade 3 events attributable to the agent. Therefore it may be concluded that BC-819 is safe to use intraperitoneally in patients with ovarian, peritoneal and tubal cancer .Since the study is limited by the small number of patients, future studies should include larger cohorts, higher doses, longer periods of treatment and combination with other systemically administered drugs.
